# Effects of intimate partner violence against women in international micro and small enterprises relationships: The mediator role of capabilities

**DOI:** 10.3389/fpsyg.2022.950807

**Published:** 2022-10-04

**Authors:** Jazmín E. Ponce-Gómez, Arístides Vara-Horna, Alba Valenciano-Mañé, Utz Dornberger

**Affiliations:** ^1^Small Enterprise Promotion and Training Competence Center, Leipzig University, Leipzig, Germany; ^2^Faculty of Management and Human Resources, University of San Martin de Porres, Lima, Peru; ^3^Social Anthropology Department, Autonomous University of Madrid, Madrid, Spain

**Keywords:** international trade, gender, export, capabilities, relationship quality, violence against women, women owned enterprises, women exporters

## Abstract

While previous studies have explored multiple constraints affecting women exporters, the effects of intimate partner violence against women (IPVAW) are still unexplored in the literature. Thus, this study aims to probe first whether women owners of micro and small enterprises (MSEs) in export markets experience IPVAW. Secondly, it aims to explore the effect of IPVAW on their relationship quality with business partners, mediated by the performance of export capabilities. Using a structured questionnaire applied through personal interviews, we surveyed 57 female owners of exporting MSEs in Peru. Partial least squares structural equation modeling was used to analyze the data and test the model. The preliminary findings indicate that women exporters who experience IPVAW are more likely to face problems performing export capabilities. In turn, these performance problems seem to affect the quality of their relationships with importers and suppliers. Moreover, there is a direct relationship between IPVAW and problems in performing export capabilities and between issues performing capabilities and the relationship quality. Our theoretical contribution is a conceptual model that proposes the variable “Problems Performing Capabilities” as a mediator to measure the effects of IPVAW on exporting MSEs owned by women. Our findings urge policymakers and trade organizations boosting women-owned export enterprises to include initiatives that address and prevent IPVAW in their export promotion programs.

## Introduction

Boosting the participation of women entrepreneurs in international trade makes countries’ economies more competitive (Kabeer, 2012; International Trade Centre, 2015) and contributes to the advancement of sustainable development goals. In this mission to potentiate women-owned export businesses, various reports have explored business constraints they face in export markets (e.g., Orser et al., 2004; Brenton et al., 2011, 2013; Lee et al., 2016; Pozarny, 2016; Welch et al., 2016; Rosenbaum, 2017, 2019; Asia-Pacific Economic Cooperation Secretariat, 2017; Shepherd and Stone, 2017; World Bank, 2020; Davies and Mazhikeyev, 2021).

Nevertheless, we cannot discuss women entrepreneurs’ constraints in export markets without including the gender issues affecting them. One of the most considerable gender constraints for women around the globe that has not been considered until now by the literature is intimate partner violence against women (IPVAW). IPVAW violates women’s human rights and is a continuous pandemic even older than COVID-19. Worldwide, one in three women has experienced intimate partner violence and non-partner sexual violence at least once in their lifetime (World Health Organization, 2021). IPVAW does not only compromise women’s wellbeing and causes tremendous costs for growth and development (Varcoe et al., 2011; Duvvury et al., 2013; Vyas, 2013), but it also affects the work productivity of owners of micro-enterprises in local markets (Vara-Horna, 2015, 2018, 2020a). On this previous evidence, it is not unwise to hypothesize that IPVAW could also affect women owners of micro and small enterprises (MSEs) that operate in export markets.

To date, it is unknown whether women-owned export businesses experience IPVAW. Besides, no study has investigated the effects of IPVAW on their export businesses. Indeed, there is a current lack of knowledge regarding the prevalence and effects of IPVAW on international women entrepreneurs. This study is the first to fill this knowledge gap by answering two fundamental questions: (1) do women owners of exporting MSEs experience IPVAW? And if so, (2) how does IPVAW affect their exporting MSEs? To answer these questions, we conducted an exploratory study with two aims: (1) to evidence that women owners of exporting MSEs experience IPVAW, and (2) to explore the effects of IPVAW on their exporting MSEs by proposing and testing a conceptual model.

This study proposes a conceptual model building on the resource-based view theory of the firm (RBV) applied to export businesses and previous evidence on the effects of IPVAW. Our model analyzes the effects of IPVAW on the relationship quality with importers and suppliers and four types of crucial exporting capabilities that enhance the export performance of small businesses. In this model, we acknowledge that the owner plays a crucial role in achieving export performance, as they are mainly responsible for making management decisions (Celec and Globocnik, 2017), obtaining resources, and performing capabilities to export. We tested the proposed model with 57 women owners-managers of exporting MSEs in Peru, one of the countries with the highest levels of physical and sexual intimate partner violence in Latin America and the Caribbean (Bott et al., 2021). As a result, we provide the first evidence that IPVAW increases the probability of women owners having problems performing capabilities, affecting the relationship quality with importers and suppliers.

The research findings of this study offer a series of conceptual and practical contributions. First, it enriches the literature on female international entrepreneurship by revealing for the first time that IPVAW is a constraint that affects women owners of exporting MSEs and their businesses. Secondly, it provides and tests a first conceptual model to measure the effects of IPVAW on women owners of exporting MSEs and proposes the variable “problem performing capabilities” as a mediator variable. Finally, the findings provide evidence for policymakers and trade promotion organizations to design export promotion programs for women-owned businesses that include measures to prevent, assess, and combat IPVAW. Addressing IPVAW will potentiate their actions’ effectiveness and tackle the constraints affecting women exporters in a better scope.

The article is organized as follows. Preceding the introduction described below, we present the proposed model to measure the effects of IPVAW together with our hypotheses. Afterward, we explain the research methodology, the research results, and a discussion of the findings. Finally, we conclude with theoretical and practical implications, limitations, and future research directions.

## Conceptual model

**Figure 1:**
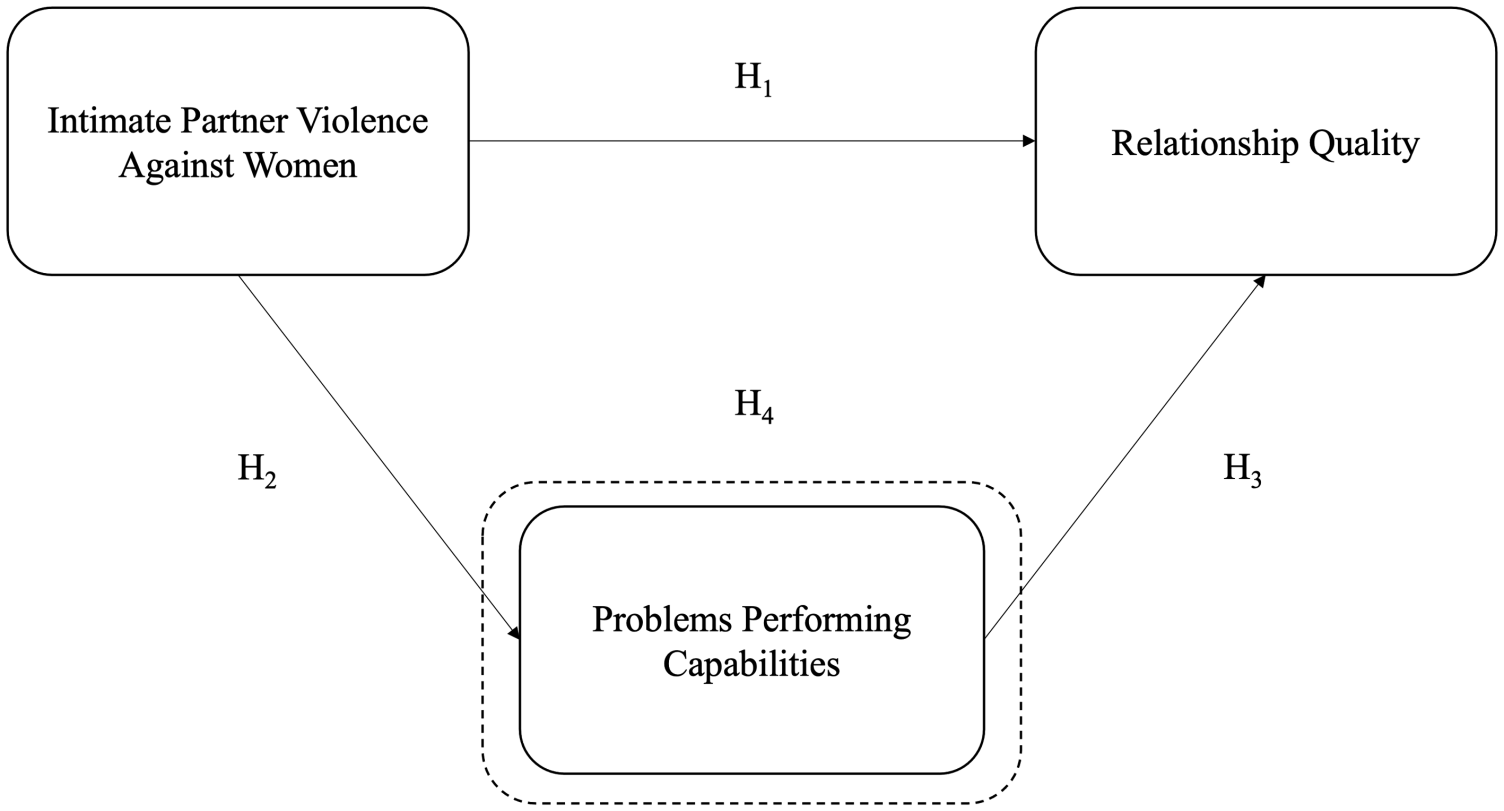
Conceptual framework and hypotheses. The dotted outlining represents the mediating variable.

The first and crucial hypothesis of this study is that women exporters are experiencing IPVAW in any of its forms, psychological, economic, physical, or sexual violence. We start from this affirmation to propose a conceptual model that measures the effects of IPVAW on women-owned exporting MSEs. Since there is no evidence in the literature about the effects of IPVAW on this group of women entrepreneurs, there is a need to create a conceptual model. Thus, we propose a model nourished by literature on the resource-based view (RBV) theory of the firm applied to small and medium enterprises (SMEs) in export markets, together with previous evidence about the effect of IPVAW on female employees and women owners of micro-enterprises. The conceptual model presented in Figure 1 seeks to integrate that evidence.

To measure the effect of IPVAW on women-owned exporting MSEs, we used evidence from the RBV theory of the firm applied to the SMEs’ performance in export markets. The RBV states that the firm’s performance and competitive advantage result from effectively using its resources and capabilities (Wernerfelt, 1984; Barney, 1991; Makadok, 2001). In the context of international SMEs, plenty of literature has examined the positive effect that a set of resources and capabilities have on the performance of SMEs in export markets (e.g., Kaleka, 2002; Zou et al., 2003; Morgan et al., 2004; Lages et al., 2009; Celec and Globocnik, 2017; Ferreira and Simões, 2017; Monteiro et al., 2019; Gupta and Chahuan, 2021). Nonetheless, not all factors are critical for superior export performance. For example, special attention has been brought to relational resources or the relationship quality with business partners (Davis and Mentzer, 2008; Monteiro et al., 2019; Leonidou et al., 2021).

Relational resources refer to the ties between the firm and external stakeholders like customers, suppliers, and others like government institutions and competitors (Davis and Mentzer, 2008). The quality of these relations–relationship quality–is commonly measured by the punctuality, trust, quality of communication, and commitment between the business partners (Kuhlmeier and Knight, 2010; Leonidou et al., 2013, 2014, 2021; Bianchi and Abu Saleh, 2020). As found by diverse authors, strong and positive relationships with business partners and foreign customers positively influence SMEs’ export performance (Lages et al., 2009; Ural, 2009; Celec and Globocnik, 2017; Haddoud et al., 2017; Hasaballah et al., 2019; Monteiro et al., 2019; Imiru, 2022). Indeed, good relationships with importers result in a better flow of information about the target market, customer needs, and competitors, allowing exporters to improve before and after-sales services, develop new products, and design better costs and pricing strategies (Ambler and Styles, 2000; Zou et al., 2003; Beleska-Spasova et al., 2012; Monteiro et al., 2017, 2019).

Given that small enterprises have limited resources developing positive relationships with foreign partners plays a crucial role in competing in international markets (Knight and Cavusgil, 2004; Lages et al., 2009). The situation is not different for women exporters, whose enterprises are mainly small and tend to have limited resources to compete in international markets (Frohmann, 2018). Therefore, close relationships with customers and suppliers are a high priority for some women exporters to earn a reputation and business deals (Reavley et al., 2005). In this context, the question is how IPVAW could affect these crucial relationships. Since no study explored the effects of IPVAW on the relationship quality with business partners, we considered a previous study showing that women micro-entrepreneurs who experienced IPVAW had a higher prevalence of problems with their customers than those who did not suffer IPVAW (Vara-Horna, 2015, 2018). Although these studies were based on a prevalence comparison between the group of women with and without IPVAW, they gave us the insight to hypothesize that IPVAW could affect the relationship of women exporters with business partners such as importers and suppliers. Therefore, our first hypothesis states:

*H1*: IPVAW has a direct effect on the quality of the relationship between women exporters with importers, and suppliers

Moving forward with our model, possessing resources is insufficient to develop a competitive advantage and ensure export success. Following the RBV theory, the performance of capabilities is needed to differentiate from competitors and offer value to export markets. The literature suggests four particular types of capabilities influencing the export performance of SMEs: operational, informational, product-development, and relationship-building capabilities (e.g., Piercy et al., 1997; Kaleka, 2002; Morgan et al., 2004; Fernandes et al., 2017; Ramon-Jeronimo et al., 2019; Aghazadeh et al., 2022). We consider these four capabilities together as export capabilities.

Operational capability is the ability to exploit resources (Nonaka, 1991; Bromiley and Rau, 2014) to perform the enterprise’s basic functional or daily activities (Winter, 2003; Pavlou and El Sawy, 2011). In the context of exports, these are basic critical activities needed to export, such as obtaining export documentation like certificates, compliance with quality standards, and compliance with the legal export procedures and regulations to export (Sharma, 2013). Informational capability is acquiring relevant information about the international market, customers, competitors, and the export market environment (Souchon and Diamantopoulos, 1996) to make better decisions and perform better (Morgan et al., 2004). This capability provides a competitive advantage in export markets (Kaleka, 2002) and is positively related to the SME’s export performance (Kaleka, 2012; Celec and Globocnik, 2017; Keskin et al., 2021). For others, it is considered an export success factor (Ferreira and Simões, 2017; Monteiro et al., 2019). The third capability is Relationship-building, which refers to establishing and maintaining close business relationships with customers and suppliers (Kaleka, 2002; Morgan et al., 2004). Building good and lasting relationships, especially with international customers, is essential to achieving a competitive export advantage and improving export performance (Kaleka, 2002; Ramon-Jeronimo et al., 2019; Malca et al., 2021). It also includes negotiation skills (Atkin and Rinehart, 2006), as a coercive or non-cooperative negotiation can negatively affect the negotiation’s success perception and, ultimately, their relationship. Finally, Product-development capability means the development of new products, improvement, modification, or adaptation of existing ones, and adoption of new methods and ideas in the production process (Morgan et al., 2004; Ferreira and Simões, 2017; Ramon-Jeronimo et al., 2019) to satisfy the needs of international customers (Zou et al., 2003). The performance of this capability provides a competitive advantage and creates and delivers superior value to international customers (Zou et al., 2003; Morgan et al., 2004; Aghazadeh et al., 2022).

Previous reports have also highlighted the relevance of these capabilities for women exporters (Brenton et al., 2013; International Trade Centre, 2015; Pozarny, 2016; World Bank, 2020). In this scenario, IPVAW could affect women exporters’ performance in the export capabilities described below. For instance, previous research in Bolivia, Peru, Ecuador, and Paraguay found that IPVAW has diminished the work productivity of female employees, who had more problems concentrating at work and difficulties fulfilling their usual tasks (Vara-Horna, 2019, 2020b). Similarly, local women entrepreneurs who suffered IPVAW have diminished their work productivity and lost working days compared to women without IPVAW (Vara-Horna, 2015, 2018, 2020a). With these antecedents, we hypothesize that women exporters who experience IPVAW will likely face more problems performing export capabilities. Hence, we propose the following hypothesis:

*H2*: IPVAW directly affects women exporters' problems performing export capabilities.

Nevertheless, based on the RBV theory, resources and capabilities are interrelated (Morgan et al., 2004), meaning that the resources could leverage the development of capabilities and vice versa (Morgan et al., 2004; Wu, 2006; Lin and Wu, 2014). The performance of organizational capabilities, such as market, learning orientation, and innovativeness, positively influences the quality of the relationships with suppliers and, consequently, enhances the business performance (Chong Tan et al., 2011). For exporting SMEs, the performance of some competencies, such as managerial, positively influences the relationship quality with importers, which, in turn, affects its competitive advantage (Ismail et al., 2014). While these authors analyzed different capabilities, we hypothesize that if the owner has problems performing export capabilities, the relationship quality with importers and suppliers could be affected. Initially, without including the effects of IPVAW, we hypothesize that women exporters’ poor performance of export capabilities could affect the relationship quality with importers and suppliers. Hence, we formulate the following hypothesis:

*H3*: Women exporters' problems performing export capabilities have a direct negative effect on their relationship quality with importers and suppliers

Finally, building on the hypothesis above described and previous evidence on the effects of IPVAW on local women micro-entrepreneurs, our model proposes a potential relationship between IPVAW, export capabilities performance, and the relationship quality with business partners. We hypothesize that women owners of exporting MSEs experiencing IPVAW could have problems or difficulties performing export capabilities, which could affect their relationship quality with importers and suppliers. Our model situates the variable “problems performing export capabilities” as a mediator between IPVAW and the relationship quality with the business partners. Therefore, the last and central hypothesis of this study is:

*H4*: IPVAW negatively affects women exporters’ relationship quality with business partners, mediated by women’s problems performing capabilities.

## Methodology

### Research design

This study uses an exploratory approach since it is the first study exploring the effects of IPVAW on exporting MSEs owned by women. While the instrument used during the data collection was a structured questionnaire, we conducted personal in-depth interviews with women exporters to gain the respondents’ trust and clarify the questions.

### Participants and data collection process

In outlining the population of women exporters in Peru, we discovered that there were no statistics, directories, or official databases of women-owned export enterprises that could give us a reference for the population, their location, or the sector’s participation. Thus, there was the need to build an official directory of women-owned export MSEs collecting and crossing official databases from three different national institutions: the National Superintendence of Customs and Tax Administration (SUNAT), the Ministry of Production, and the Comprehensive Foreign Trade Information System (SIICEX). In the first stage, we requested the database of all enterprises with exports in 2018 and 2019, their location, and the owner’s gender. Surprisingly, information about the owner’s gender was unavailable in the national statistical system. Therefore, we could only access the owners’ names of all enterprises. With this, we built up a database of MSEs with three criteria: (1) The enterprise was categorized as MSE according to the Peruvian legislation. Their total annual sales were not more than S/0.7,140,000 soles or US$2,106,194. (2) The MSEs exported in 2018 and 2019, signifying that they are not sporadic exporters but have 2 years of exporting knowledge and experience; and most importantly, and (3) The MSEs were managed and owned by women at an equal or greater percentage than 50%.

In the second stage, we conducted a desk validation, tracking the identification number of each enterprise and verifying that they were active by December 2019. We reviewed the official status of all companies in the official national online system. Companies with the status of suspended, not locatable, or in liquidation were not discarded. After that, we compiled the companies’ telephone and e-mail available on diverse web pages. As a result of the desk validation, Lima and Arequipa were the regions with more women exporters in Peru.

In the third stage, we made phone calls to all the MSEs from the filtered database to confirm that the enterprises were currently operating (active) and to verify that the owners and general manager of the company were women. Nevertheless, the telephone numbers were not working most of the time, and the owner was unavailable. Therefore, we sent invitations to all the enterprises’ e-mails that we compiled with an invitation to participate in this research.

We called and sent e-mail invitations to 228 MSEs in Lima, of which 146 did not answer e-mails or phone calls. We contacted 82 women exporters by phone, of which 39 did not agree to participate in this study. In Arequipa, we called and sent invitations to 25 eligible companies, from which three did not answer. Five refused to participate due to the lack of time or availability. We arranged personal meetings with the women owners at their enterprises’ premises or cafeterias near their workplaces. Only two interviews were conducted by video call due to the COVID-19 outbreak in Peru.

As a result, we interviewed and surveyed 60 women from Lima (43) and Arequipa (17). Nevertheless, one survey in Lima and two in Arequipa were invalid because interviews were interrupted and questionnaires could not be completed. In total, we collected answers from 57 women owners of exporting MSEs. They were, at the same time, the general managers of the exporting MSEs. Each interview to administer the questionnaire lasted between 40 and 60 min.

The empirical material was collected through a structured survey using an interview technique to gain the respondents’ trust and confidence. The data collection period was from January to March 2020. First, we started the interviews by briefly introducing the research. Then, we informed the anonymity of their answers and requested their consent to start the interview. During the interview, we read every question and filled out the survey according to their answers.

In some cases, the interviewees provided more details about their experiences running their exporting enterprises and their experiences with IPVAW. As suggested by Ellsberg and Heise (2007), we applied an ethical protocol that assured the informants’ confidence, security, anonymity, and protection. If someone approached the informants during the meeting, we changed the conversation topic and did not mention IPVAW. At the end of the meeting, we offered information about support lines and current public services that addressed IPVAW.

### Questionnaire and measurements

The questionnaire was validated in two stages. Firstly, by a group of experts on IPVAW and its effects on businesses. Secondly, we ran a pilot survey with 10 women exporters to try the easiness of the questions and improve the questions’ content. This questionnaire included items related to five themes: (1) General demographic information, (2) MSEs’ export activity, (3) Relational resources, (4) Problems performing export capabilities, and (5) IPVAW. In addition to demographic information of the women owner and the MSEs’ export activity, three main variables were measured:

#### Relationship quality

A 12-item scale measured the relationship quality of the women exporter with importers that are (1) customers (final consumers or companies that use the product as input for manufacture or transformation) and (2) distributors (clients that resale the product). Moreover, we included the relationship quality with suppliers (local or international that provide them the final product or input for production). The quality of the relationship was measured by analyzing four dimensions: punctuality, trust, quality of communication, and commitment (Kuhlmeier and Knight, 2010; Leonidou et al., 2013, 2014). The constructed scale was created using items with the highest reliability from the dimensions developed by Kuhlmeier and Knight (2010) and Leonidou et al. (2013). To answer the questions, women exporters evaluated how they think customers/distributors/suppliers have perceived them in the last year in terms of punctuality, trust, communication, and commitment. The response options were a 7-point Likert scale ranging from unpunctual to very punctual (punctuality), not at all reliable to reliable (trust), poor and limited communication to fast and relevant communication (communication), and without commitment to very committed (commitment).

#### Problems performing export capabilities

This construct measured the number of times women entrepreneurs faced problems or difficulties performing four types of export capabilities in the last 12 months. Each item is measured with a 7-point Likert scale developed by Vara-Horna (2015; 2020a) with ordinal response alternatives for the number of incidents: never; happened only before 2019, one or two times; 3–5 times, 6–10 times; between 11 and 20 times; and more than 20 times.

##### Operational capabilities

Five-item scale measured if women exporters face problems or difficulties performing operational activities or procedures entirely necessary to export (Katsikeas and Morgan, 1994), such as (1) obtaining documentation to export like certificates (origin certificate and specialized certificates according to the product), (2) compliance with quality standards requested by the clients, (3) compliance with the legal export procedures and regulations to export, and (4) achievement of the delivery of products in the requested times.

##### Informational capabilities

A four-item scale adapted from Celec and Globocnik (2017), Kaleka (2002), and Morgan et al. (2004) measured if women exporters had problems obtaining information such as (1) identification of potential customers, (2) capturing important market information, (3) in acquiring relevant information, and (4) in making contacts in the export market.

##### Relationship-building capabilities

A five-item scale measured problems or difficulties: (1) understanding overseas customer requirements, (2) establishing and maintaining close business relationships with customers, distributors, and suppliers. These items were obtained from Morgan et al. (2004), Ferreira and Simões (2017), and Ramon-Jeronimo et al. (2019). We included the item *“Negotiation with international clients*” after the validation with experts.

##### Product-development capabilities

A three-item scale measures problems or difficulties on: (1) the improvement or modification of existing products according to international requirements developing new products, (2) the development of one or more new products for international markets, and (3) the adoption of new methods and ideas in the production/manufacturing process. Items from this scale were used by Morgan et al. (2004) and Kaleka (2002).

#### Intimate partner violence against women

The last variable measures the presence of violent acts perpetrated by the present or former partner (boyfriend, fiancé, spouse, and partner) against the participants. These violent acts are typified by psychological, economic, physical, and sexual and are measured on an additive scale. The 20-item-scale was adapted from Vara-Horna (2015, 2019, 2020a), the Conflict Tactics Scale—CTS 2 (Straus, 2007), and the national IPVAW survey of the Center for Disease Control and Prevention (Saltzman et al., 2002). After conducting the surveys, we excluded three items that did not have prevalence: two items that measured sexual violence and one that measured physical damage as a consequence of violence. All items included in the analysis are listed in Table 3. IPVAW scales showed internal consistency and construct and discriminant validity. The average extracted variance by scale ranged between 87.8% and 91.8%, with high composite reliability (between 0.845 and 0.957) and discriminant validity (Vara-Horna, 2019). Each item has ordinal response alternatives for the frequency of IPVAW that range from never; it happened only before 2019; one or two times; 3–5 times, 6–10 times; between 11 and 20 times; and more than 20 times. In addition, the scale differentiates two time periods: (a) IPVAW last year: any act of IPVAW occurred within the last 12 months (the year 2019) and (b) IPVAW during lifetime: IPVAW occurred in the last year and in before the last year.

### Participants description

Fifty-seven Peruvian women owners and managers of exporting MSEs from the regions of Lima and Arequipa participated in this research. The average age of the women exporters was 46 years, ranging from 19 to 76 years. According to the educational level, more than half of women exporters have a graduate or postgraduate degree (64.7%). Most women speak a second language (82.4%), principally English (80%), and French (20%). The majority had an intimate partner (63.2%) and a son or daughter (71.9%). According to their firm’s information, their companies have 12 years of creation on average, ranging from 2 to 40 years. Their economic sector is principally the textile sector (45.6%), followed by agro-export (8.8%), Handicrafts (8.8%), Metal-Mechanical (7%), Food and Beverages (7%), and other sectors. Only 24.6% of women exporters were unique owners, while 75.4% had a business partner, mostly family members (47.7%) and an intimate partner (20.5%). Nevertheless, all women interviewees oversaw the management of the enterprise. As per the full-time employees, the majority had 1–10 employees (61.4%) and 11–20 employees (17.5%). While 8.8% had between 21 and 60 employees and 12.3% had no employees. Considering the employees involved in export management, half of the women have at least one employee that supports them (50.9%), and 10% have the support of two to four employees. However, 38.6% manage the export activities alone.

### Data analysis

To analyze the data and test the model, we applied partial least squares structural equation modeling (PLS-SEM) using the statistical package SmartPLS 3.3.2. The reliability and validity of the scales were optimal. The Cronbach’s Alpha value ranges from 0.751 to 0.866, and the Average Variance Extracted is more than 50% (see Table 1).

**Table 1:** Reliability and validity of the constructs.

Constructs	Alfa de Cronbach	rhoA	Composite reliability	Average variance extracted
Intimate partner violence against women	0.751	0.787	0.813	0.539
Problems performing capabilities	0.760	0.719	0.744	0.521
Relationship quality	0.866	0.793	0.865	0.636

We consider the PLS-SEM approach the most suitable method to test the model for various reasons (Hair et al., 2019). First, PLS-SEM is a non-parametric method that does not require normally distributed data. In this regard, we found that the constructs were not normally distributed after running Kolmogorov–Smirnov and Shapiro–Wilk tests into our data. Therefore, we needed a non-parametric test to analyze the correlation between variables. Second, PLS-SEM is used in exploratory research to discover new relationships, develop a theory, or explore the extension of existing ones (Hair et al., 2019). Fourth, PLS-SEM has been increasingly used to improve the explanatory capacity of variables and their relationships (Hair et al., 2014; Matthews et al., 2018), especially in social and behavioral sciences (Benitez et al., 2020) and international business research (Richter et al., 2016). Indeed, this research is exploratory and aims to detect a relationship between IPVAW, Relationship quality with business partners, and women’s problems performing capabilities which other studies have not proved. Finally, PLS-SEM is used when the model’s structure is complex and analyzes the relationships considering a mediator variable (Henseler et al., 2016; Matthews et al., 2018). Indeed, the proposed model aims to explore the mediation effect of the variable “problem performing capabilities” to prove the effects of IPVAW.

The discriminant validity was estimated using the Heterotrait-Monotrait (HTMT). According to Henseler et al. (2015), the HTMT criterion is more precise in measuring discriminant validity as it can achieve superior specificity and sensitivity rates (97–99%) in comparison to the Fornell–Lacker criterion and cross-loadings (Henseler et al., 2015). In addition, the HTMT criterion states that the constructs’ independence is confirmed when the values are less than one, meaning that there is no redundancy. In contrast, values close to one mean a lack of discriminant validity. To use the HTMT, some authors recommend a threshold of 0.85 or 0.90 (Fornell and Larcker, 1981; Kline, 2015). As observed in Table 2, all HTMT values are below one and the suggested threshold, meaning that the constructs have discriminant validity.

**Table 2:** Heterotrait-Monotrait ratio—HTMT for discriminant validity.

Construct	IPVAW	Problems performing capabilities	Relationship quality
IPVAW			
Problems performing capabilities	0.689		
Relationship quality	0.400	0.488	

A bootstrapping method was implemented to examine the trajectory of beta coefficients and determine if these relationships are significant. Bootstrapping is a resampling technique that creates artificial parameters. It reproduces 5,000 times the analysis and all indicators obtained by combining the data from the 5,000 analyses. This technique estimates the standard errors to calculate the Student’s *t*-values and the significance of the Beta coefficients (significance value of *p*). The relationships are considered significant if *p* < 0.05 and *T* is greater than the critical value (1.96, with a significance level of 5%; 2.57, with a level of significance of 1%) (Hair et al., 2014, 2017). As control variables, non-statistical differences were found between the group of women who experienced IPVAW and who did not experience IPVAW in terms of age (*F* = 0.005, *p* = 0.994), educational level (*F* = 1.221, *p* = 0.275), precedence from Lima or Arequipa (*X*^2^ = 0.243, *p* = 0.073), civil status (*X*^2^ = 7.03, *p* = 0.218) and age of the business (*F* = 0.003, *p* = 0.995).

## Results

Of all women exporters, 61.4% of women exporters have experienced IPVAW at least once in their lives, and 40.4% in the last 12 months (see Table 3). The types of violence with the highest prevalence were psychological (61.4%), physical (17.5%), and economic violence (10.5%). Psychological violence is the most common type of IPVAW experienced in 2019 (40%). None of the interviewed women experienced sexual violence by their partners or former partners.

**Table 3:** Prevalence of women exporters who suffered IPVAW by their partner or former partners.

	Prevalence	
	Life (%)	Last 12 months (%)
Psychological violence	61.4	40.4
Has he humiliated you, making you feel bad about yourself	43.9	26.4
Has he harassed you while you were working or while you were not at home	21.1	8.8
Has he forbidden you, through warnings or threats, to chat with friends, clients, or other men	10.5	5.3
Has he forbidden you, through warnings or threats, from traveling for business	8.8	5.3
Has he threatened to kill himself if you leave him or hurt you and your family	10.5	1.8
He has insulted you verbally, said rude or aggressive words	43.9	28.1
Economic violence	10.5	1.8
Has he forced you to obtain credit against your will	1.8	0
Has he taken your money, personal belongings, or property away from you	7	1.8
Has he destroyed your belongings, clothes, or documents	5.3	0
Has he taken money, destroyed supplies, products, or documents from your business	1.8	1.8
Physical violence	17.5	0
Has he slapped you or pulled your hair	8.8	0
Has he pushed you against the wall or floor	12.3	0
Has he punched or kicked you	3.5	0
Has he hit you with a leash, sticks, or other objects	0	0
Has he tried to strangle you	3.5	0
Has he attacked or threatened you with sharp weapons (knife) or guns	1.8	0
Total IPVAW	61.4	40.4

A summary of the annual and lifetime prevalence of the difficulties performing export capabilities is presented in Table 4.

**Table 4:** Prevalence of problems performing export capabilities.

	Prevalence	
	Life (%)	Last 12 months (%)
Operational capabilities		
Obtain certificates or other documentation for export	68.6	47.1
Comply with quality standards or requirements of the export product	35.1	21.1
Comply with export procedures and regulations	36.8	26.3
Achieve the delivery of the orders in the requested times	63.2	49.2
Informational capabilities		
Identify potential customers	61.4	52.6
Capture/acquire important information from international markets	54.4	45.6
Make new contacts in the international market	63.2	52.6
Monitor competitive products in the international market	45.6	35.1
Relationship-building capabilities		
Negotiate with your international customers	50.3	32.8
Understand the requirements of your international clients	40.4	28.1
Establish and maintain close relationships with your international customers (who are not distributors)	22.8	14
Establish and maintain close relationships with your international distributors	33.3	21
Establish and maintain close relationships with your suppliers	42.1	19.3
Product-development capabilities		
Improve/adapt your existing product(s) to international requirements	45.6	29.8
Develop one or more new product(s) for international markets	45.6	36.8
Adopting new methods and ideas in the production/manufacturing process	42.1	38.8

Applying partial least squares (PLS) for structural equations, we tested our model and explored the direct, indirect, and total effects of IPVAW on the relationship quality and export capabilities performance (see Table 5). The analysis shows that IPVAW has no significant direct impact on the relationship quality with business partners, rejecting Hypothesis 1 (*β* = −0.120; *p* = 0.508). In contrast, IPVAW has a positive direct relationship with women’s problems performing export capabilities (*β* = 0.460; *p* < 0.01), confirming Hypothesis 2.

**Table 5:** Hypotheses testing results.

Hypotheses	Path	Beta	t	Confidence interval 95%		Result
H1	IPVAW > RQ	−0.120	0.662	−0.571	0.141	Rejected
H2	IPVAW > PPC	0.460	3.041**	0.332	0.778	Approved
H3	PPC > RQ	−0.436	2.992**	−0.715	−0.167	Approved
H4	IPVAW > PPC > RQ	−0.200	2.012*	−0.442	−0.070	Approved

PPEC, problems performing capabilities; IPVAW, intimate partner violence against women; RQ, Relationship quality. *p < 0.05; **p < 0.01.

Moreover, we found that women’s problem performing capabilities negatively affect the relationship quality with business partners (*β* = −0.436; *p* < 0.01), supporting Hypothesis 3.

We tested our model and confirmed Hypothesis 4. IPVAW indirectly affects the relationship quality of women’s business partners, mediated by the problems performing export capabilities (*β* = −0.200; *p* < 0.05). In other words, women owners of exporting MSEs who experience IPVAW have more probability of facing problems performing capabilities, which tends to affect their relationship with business partners.

## Discussion

This study evidence for the first time that women owners of exporting enterprises suffer intimate partner violence, affecting their performance. Indeed, we tested an initial conceptual model finding that IPVAW increased the probability of facing problems performing capabilities, affecting their relationship quality with importers and suppliers. Since no study has explored IPVAW as a constraining factor for women entrepreneurs in export markets nor explored how it could affect women’s export businesses, our research makes significant theoretical and practical contributions.

Similarly to the findings of previous studies on women micro-entrepreneurs in local markets (Vara-Horna, 2013, 2015, 2018, 2020a), our study evidence that women owners of MSEs in export markets also experience IPVAW, mainly psychological, economic, and physical violence. A possible explanation of why these international businesswomen, who have a higher educational level, experience IPVAW, is because empowerment is not immediate protection against violence, as the male backlash theory predicts. As has been reported by various studies, female empowerment in terms of employment opportunities and higher educational achievement tend to increase domestic violence and abuse (Heath, 2014; Erten and Keskin, 2018, 2021; Bhalotra et al., 2021; Guarnieri and Rainer, 2021). Furthermore, even in a gender-equal country like Sweden, intimate partners tend to react with hostility and violence to an improved economic position of women (Bergvall, 2022). In this regard, women owners of exporting MSEs are not exempt from suffering IPVAW; their role as business owners and economic position could trigger a male backlash response from the partners, who react with hostility and violence against them.

While this study confirmed that IPVAW is a problem affecting women owners of exporting MSEs, we also aimed to explore the effects of IPVAW on women-owned exporting MSEs. As previously mentioned, there is no evidence on how IPVAW can affect them. Therefore, we proposed and tested a model built-up on the literature about the resources and capabilities needed by SMEs in export markets, together with evidence on the effects of IPVAW on the productivity of female workers and women micro-entrepreneurs. Our model includes the relationship quality with business partners and a set of capabilities needed by an SME to be successful in export markets. As a result, we explored the direct effects of IPVAW and indirect effects of IPVAW on the relationship quality with importers and suppliers, mediated by women exporters’ problems performing capabilities.

Our first hypothesis explored if IPVAW directly affects the relationship quality with importers and suppliers. After analyzing the data, we found that IPVAW does not have a direct effect on the relationship quality, rejecting hypothesis 1. With this result, it seems IPVAW does not affect the relationship quality of women exporters with these business partners. However, this analysis was without considering a mediator variable. Previously, Vara-Horna (2015, 2018) suggested that IPVAW could be related to the relationship of women micro-entrepreneurs. However, these studies on micro-entrepreneurs did not aim to evaluate the effect of IPVAW and the relationship with clients but exposed that women with IPVAW had a higher prevalence of problems with customers compared to the women that did not suffer IPVAW. In comparison, our analysis explored the direct effect of IPVAW on the relationship quality of women exporters with business partners.

In terms of the effect on capabilities, we found that IPVAW has a direct effect on women’s problems performing export capabilities. In other words, women exporters that experienced IPVAW had a higher probability of facing problems performing export capabilities. This finding is consistent with previous studies evidencing that female workers and owners of local microenterprises who experienced IPVAW were more likely to have a diminished work performance, being less concentrated, and motivated at work compared to those who do not suffer from IPVAW (Vara-Horna, 2015, 2018).

Without considering the IPVAW variable, we aimed to evidence a relationship between the problem performing capabilities and the relationship quality with importers and suppliers. Our findings confirm a direct relationship between women exporters’ export capabilities and the relationship quality with importers and suppliers. This goes in line with previous studies, where the performance of capabilities enhanced the quality of the relationship with customers and suppliers (Chong Tan et al., 2011; Ismail et al., 2014).

Building on this relationship, we probe our model when the variable *problems performing capabilities* is a mediator between IPVAW and relationship quality. In other words, women exporters who experience IPVAW are more likely to face problems performing export capabilities. These performance problems tend to affect their relationship quality with importers and suppliers. Contrary to our proposed model, previous studies used morbidity as a mediator variable to estimate the effect of IPVAW on labor productivity (Vara-Horna, 2013, 2015, 2016, 2018; Asencios-Gonzalez et al., 2018; Duvvury et al., 2020). Nevertheless, these studies concentrated on female employees of medium or big firms, women owners of formal and informal enterprises, or women surveyed in their households. These groups had a higher annual and monthly prevalence of all four types of violence, psychological, physical, economic, and sexual. They also had severe physical damage due to violence. However, in the case of women exporters, our study found that the most declared type of violence at an annual prevalence was psychological, and no one had physical damage due to violence. In this regard, our study suggests that the variable *problem performing capabilities* could be potentially used as a mediator variable to estimate the effects of IPVAW on women’s businesses when women entrepreneurs have mainly suffered psychological violence.

### Theoretical contribution

While the literature on female entrepreneurship is vast, the stream of international female entrepreneurship remains in its developing stage. Our research enriches the literature on international female entrepreneurship and contributes to the body of knowledge in two ways. First, we evidence that women entrepreneurs in export markets are affected by IPVAW. A constraint that was not explored by any study on women exporters. Secondly, our most important theoretical contribution is the proposal of the first conceptual model to estimate the effects of IPVAW on women-owned exporting MSEs. Our research provides the first evidence that women exporters affected by IPVAW are more likely to have problems performing export capabilities, affecting the relationship quality with their importers and suppliers. We tested our model and proposed that the variable “*problem performing export capabilities*” has a mediator role in estimating the effects of IPVAW on the relationship quality with business partners. This finding opens the discussion to include this new mediator variable to measure the effect of IPVAW when women entrepreneurs have mainly suffered psychological violence. Future research should continue exploring this mediator variable to estimate the effects of IPVAW on their businesses. Our findings are an initial step for future research on international female entrepreneurs to continue testing the conceptual model in other countries’ contexts with higher or lower prevalence of IPVAW, in a specific productive sector, or even expanding and adapting the model. Overall, our study opens new directions for future investigation on the inclusion of IPVAW as a constraint for women exporters.

### Practical contribution

Our research findings are relevant for policymakers and trade promotion organizations (TPOs) aiming to empower and boost women entrepreneurs in export markets. While some have recognized a series of constraints that women exporters face and developed export support programs, IPVAW was not considered a constraint until now. We proved that women exporters face IPVAW, which negatively affects their export businesses. Our findings are an emergency call for policymakers and TPOs to destinate actions and resources to prevent and tackle IPVAW. These institutions might use our findings to act and include initiatives to address, combat, and prevent IPVAW in every export promotion program targeted at women entrepreneurs. For example, training programs could include topics related to self-empowerment, reflection on traditional gender roles, prevention of IPVAW, and the legal framework against IPVAW, among others, that seek to moderate women. NGOs or civil associations with higher expertise on those topics could be great allies in running training in this thematic.

Intergovernmental organizations, such as the Asia-Pacific Economic Cooperation and the International Trade Center (2020) with the worldwide initiative SheTrades, aiming to advance the economic integration of women entrepreneurs in international trade and empower them, might also benefit from our findings. These organizations could bring awareness about the effects of IPVAW on women exporters among their partner countries and include actions to prevent and combat IPVAW in their initiatives. In doing so, they will also contribute toward achieving Sustainable Development Goal 5, “Gender equality and women’s empowerment,” and Goal 8, “Decent work and economic growth” and respond to the “Buenos aires declaration on trade and women’s economic empowerment” (ITC, 2020). Certainly, IPVAW should not be considered a private issue that remains at home but a social and business concern that must be prevented and eliminated—with the alliance of different sectors—to guarantee women’s wellbeing and the development of their export businesses.

### Limitations and future research directions

This study has some limitations and brings new opportunities for future research. First, we acknowledge that our sample size makes it difficult to generalize our results to all women owners of exporting MSEs in Peru. Indeed, obtaining a representative sample size was challenging because women exporters were geographically dispersed. Moreover, they were not members of a business union or network, and it was necessary to invest many economic resources to locate them. Therefore, further research should continue testing the model with a more significant sample of women exporters from other emerging economies and more advanced ones. For instance, this study could be replicated in pioneer countries on gender equality in international trade such as Canada (Global Affairs Canada, 2018).

Second, our study could not examine the effects of IPVAW on the performance of each export capability and how each influenced the relationship quality with business partners. Our conceptual model proposes four key export capabilities (operational, informational, relationship-building, and product-development capabilities). Further studies could explore specific relationships between IPVAW and each export capability in robust samples. In addition, academics could use our model as a starting point and test how IPVAW affects other resources and capabilities, as suggested by the RBV theory. Finally, our study could not determine how each type of IPVAW affects women exporters and their enterprises.

Further research could inspect in depth the prevalence of the types of violence and its impact. For example, we identified that some few women exporters suffer economic violence. Academics explore further and analyze how economic violence possibly affects women exporters’ economic resources to export. Overall, our study opens a new research field in the literature on international female entrepreneurship and provides directions for further studies. We reached our aim to uncover that IPVAW is an actual constraint affecting women owners of exporting enterprises and moved one significant step in proposing and testing a conceptual model to estimate the effects of IPVAW on women-owned exporting MSEs.

## Data availability statement

The raw data supporting the conclusions of this article will be made available by the authors, without undue reservation.

## Author contributions

JP-G: contributed to the literature review, conceptualizing, performing data collection, discussion, and references. AV-H: contributed to methodology, research design, and data analysis. AV-M: contributed to conceptualizing, discussion, and overall proofreading. UD: contributed to writing and editing of the original draft. All authors contributed to the article and approved the submitted version.

## Funding

This work was partly supported by the German Academic Exchange Service (DAAD) as part of the scholarship for Development-Related Postgraduate Courses (EPOS) to JP-G during 2018–2020 (Personal Ref. No. 91705538). The publication of this paper was funded by the Open Access Publishing fund of Leipzig University supported by the German Research Foundation within the program Open Access Publication Funding.

## Conflict of interest

The authors declare that the research was conducted in the absence of any commercial or financial relationships that could be construed as a potential conflict of interest.

## Publisher’s note

All claims expressed in this article are solely those of the authors and do not necessarily represent those of their affiliated organizations, or those of the publisher, the editors and the reviewers. Any product that may be evaluated in this article, or claim that may be made by its manufacturer, is not guaranteed or endorsed by the publisher.
